# No effect of hydroxyapatite-coated sliding hip screw threads on screw migration in the femoral head/neck of pertrochanteric fractures: a randomized controlled trial using radiostereometric analysis

**DOI:** 10.1186/s13018-023-04170-0

**Published:** 2023-09-14

**Authors:** August Christoffer Krogh, Janni Kjærgaard Thillemann, Torben Bæk Hansen, Kim Holck, Morten Tange Kristensen, Henrik Palm, Maiken Stilling

**Affiliations:** 1 Department of Clinical Medicine, Aarhus University, Aarhus N, Denmark; 2 AutoRSA Research Group, Orthopaedic Research Unit, Aarhus University Hospital, Aarhus N, Denmark; 3 University Clinic for Hand, Hip and Knee Surgery, Gødstrup Regional Hospital, Herning, Denmark; 4 Department of Orthopedics, Aarhus University Hospital, Aarhus N, Denmark; 5 Department of Orthopedics, Hvidovre Hospital, Copenhagen University Hospital, Copenhagen, Denmark; 6 Department of Physical and Occupational Therapy, Copenhagen University-Hospital, Bispebjerg-Frederiksberg, Denmark; 7 Department of Clinical Medicine, University of Copenhagen, Copenhagen, Denmark; 8 Department of Orthopedics, Bispebjerg Hospital, Copenhagen University Hospital, Copenhagen, Denmark; 9 Department of Orthopedics, Palle Juul-Jensens Boulevard 165, Crossing J501, 8200 Aarhus N, Denmark

**Keywords:** Hip fracture, Pertrochanteric fracture, Radiostereometry, BoneMaster, Hydroxyapatite, Sliding hip screw, Lag screw

## Abstract

**Introduction:**

Cut-out is the most frequently reported mechanical failure of internal fixation of pertrochanteric fractures. The purpose of this study was to examine if hydroxyapatite-coated screw thread on a sliding hip screw (SHS) could reduce screw migration within the femoral head in patients with stable pertrochanteric fractures.

**Materials and methods:**

In a double-blinded randomized controlled study, 37 patients at mean age 78 (range 56–96), with pertrochanteric fracture (Evans I, II, IV) received surgery with a SHS with a hydroxyapatite-coated or a non-coated lag screw thread. Radiostereometry and standard radiographs were obtained 1 day, 6 weeks, 3- and 6 months post-operatively to evaluate screw and fracture migration and fracture reposition. The two groups were combined to describe fracture migration.

**Results:**

There was similar and small screw migration in the femoral head between the two groups at 6 weeks, 3- and 6 months (*p* > 0.12). Fracture migration occurred predominantly in the first 6 weeks, where fracture impaction was 5.95 mm (CI 95% 2.87 to 9.04) and anterior rotation of the femoral head was -2.94° (CI 95% − 5.22 to − 0.66). Migration of the fracture (total translation) correlated to the post-operative fracture reposition (*p* = 0.002), but not significantly to screw migration (*p* = 0.09). Neither screw total translation (rho 0.06, *p* = 0.79) nor fracture total translation (rho 0.04, *p* = 0.77) correlated with bone mineral density.

**Conclusion:**

There was no clinical benefit of hydroxyapatite coating on lag screw migration in this patient cohort. Migration of the pertrochanteric fractures was higher with poor fracture reposition but fractures generally stabilized after 6 weeks follow-up. The study was registered at ClinicalTrials.gov (NCT05677061).

**Level of evidence II:**

Patient-blinded prospective randomized study.

*Trial registration number* The study was registered at ClinicalTrials.gov (NCT05677061).

## Highlights

- Hydroxyapatite coating on the screw thread did not improve screw fixation in the femoral head of a sliding hip screw in pertrochanteric fractures.
- Displacement of pertrochanteric fractures operated with a sliding hip screw was primarily a fracture compression across the screw axis.
- Pertrochanteric fractures operated with a sliding hip screw stabilized after 6 weeks indicating fracture healing.

## Introduction

Hip fractures come at a high cost for the patient, the healthcare system, and for society [1, 2]. The incidence of hip fracture is increasing with the growing elderly population [3] and pertrochanteric fractures constitute a little less than half of all hip fractures in the Nordic countries [4, 5]. The principal treatment for pertrochanteric fractures is surgery with a sliding hip screw with plate (SHS), which is an implant system where a lag screw fixed in the femoral head can slide in a short barrel of a plate fixed on the lateral side of the proximal femur facilitating pertrochanteric fracture compression and healing [6–9].

The failure rate of pertrochanteric fracture fixation has been reported from 2% and up to 20% [10]. The most frequently reported mechanical failure type for different SHS devices in pertrochanteric fractures is cut-out, where the screw penetrates the cortex of the femoral head by at least 1 mm [11–13]. The cut-out complication rate of pertrochanteric fractures fixed with SHS is up to 16.7% [14]. Screw cut-out is primarily a problem in elderly patients with osteoporotic bones [15]. Optimisation of implant osseointegration has been attempted with hydroxyapatite (HA)-coated screws and have shown promising results concerning screw fixation strength [16–19]. Additionally, placement of the lag screw in the center of the head-neck axis allows both for sufficient purchase of the screw in the femoral head, which has been shown to reduce the risk of femoral head collapse into varus leading to cut-out [11, 20], and for reduction of post-operative rotation and displacement of the fracture across the trochanteric fracture line [21, 22].

Radiostereometric analysis (RSA) have proven useful, accurate and precise for three-dimensional measurement of the displacement of femoral neck fractures after osteosynthesis and for migration of orthopaedic implants in the bones [10, 23–27]. The primary purpose of this randomized clinical study was to investigate if a SHS with a hydroxyapatite-coated screw thread could reduce screw migration in the femoral head compared to a non-coated screw thread in patients with acute pertrochanteric fractures (Evans type I, II and IV). The secondary purpose was to investigate the amount of, and predictors for, rotation and translation across the trochanteric fracture line throughout a 6-month post-operative follow-up period.

## Patients and methods

**Fig. 1:**
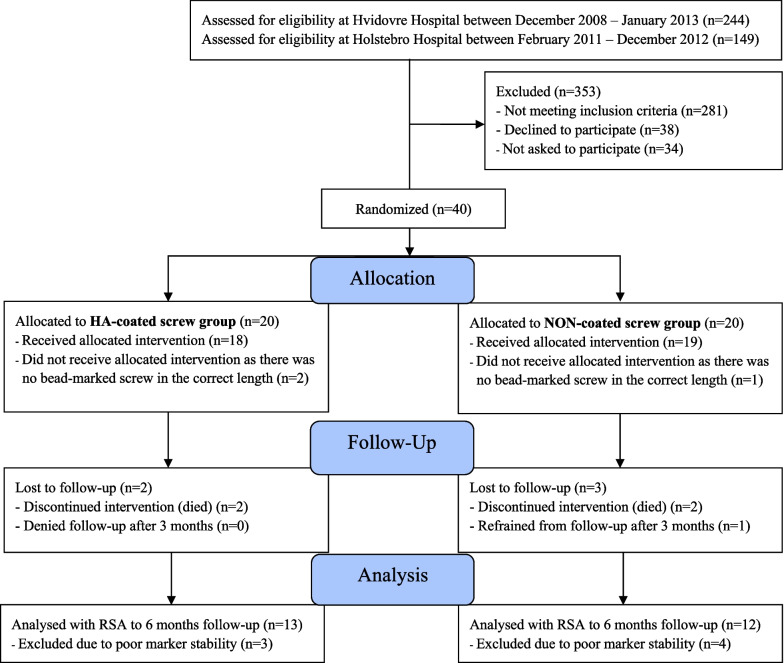
CONSORT flow chart showing the inclusion/exclusion process until 6-month final follow-up

The study was designed as a double-blinded randomized clinical trial, with blinding for patients and outcome assessors but not to the surgeons. Between December 2008 and January 2013, 37 patients were enrolled having sustained a stable pertrochanteric fracture with an intact greater trochanter and lateral femoral wall, regardless of detachment of the minor trochanter (Evans type I, II and IV). Patients were operated at two centers: Copenhagen University Hospital, Hvidovre, and Holstebro Regional Hospital, Hospital Unit West, Holstebro. Patients were randomly assigned to a lag screw with either a HA-coated screw thread or a NON-coated screw thread (Fig. 1). The randomization was performed as block-randomization (blocks of 4) by drawing labels in a box. From the randomizations list, consecutively numbered envelopes were made with information about the group allocation. After patient inclusion and anatomical fracture reposition on the operating table the next randomization envelope in the sequential order was opened and the intervention was carried out according to the allocated study group.

### Criteria

The inclusion criteria were: patients > 50 years of age admitted with a stabile pertrochanteric fracture, able to speak Danish and sign the written consent, and expected to be able to complete the postoperative controls. The exclusion criteria were: patients who were unable to follow the standard hip fracture regime, were breastfeeding, pregnant, terminal ill, in need of an open fracture reduction, in need of a SHS lateral plate with an angle different from 135° or longer than 4 holes, fractures where fewer than 3 tantalum beads were inserted in the femoral head, Tip Apex Distance (TAD) > 20 mm in two dimensions on the first post-operative X-ray, fracture displacement > 20 mm in two dimensions on the first post-operative X-ray.

### Sample size

A power analysis was performed using the y-translation screw migration measure of the first 4 patients (pilot group) after 6 months follow-up. Expecting a 40% loss of fixation with NON-coated screws [28] as compared to HA-coated screws, and using a power of 90% and alpha of 0.05 with a mean y-translation in the non-coated screws of 0.226 mm (SD 0.125) a need for 12 patients per group was calculated. Since patients with pertrochanteric fractures are generally fragile we aimed for inclusion of 40 patients (20 in each group) to balance for post-op dropouts at 6 months follow-up.

### Implants

**Fig. 2:**
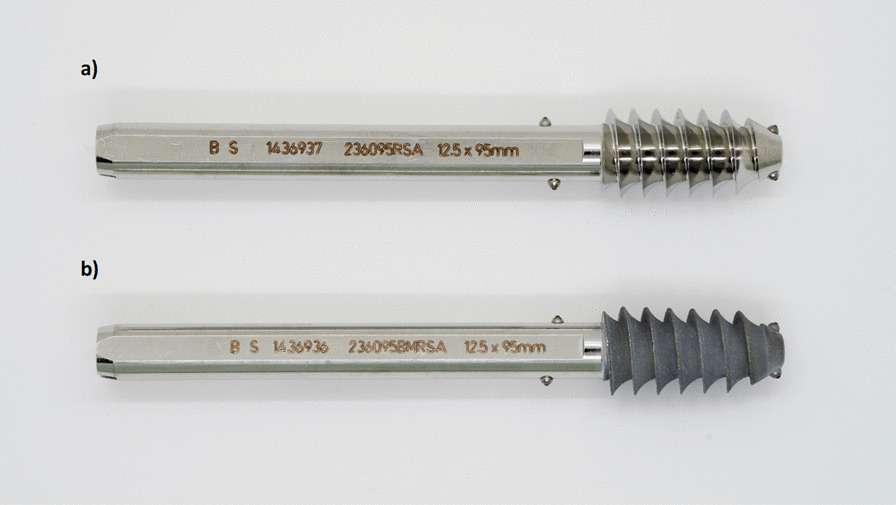
Sliding hip screw marked with 4 tantalum markers. **a** screw without coating and **b** screw with hydroxyapatite coating on the screw thread

All patients received a 4-hole 135° SHS plate (HipLOC™, Biomet, Warsaw, IN, USA). All lag screws were marked preoperatively with 4 tantalum beads in a specific predetermined pattern (Fig. 2) (Wennbergs Finmek, Gunilse, Sweden) and then packed for sterile use by Biomet. The lag screws in both groups were identical except that half of the screw threads were coated, first with plasma-spray titanium and on top of that an 5 µm electrochemically deposited hydroxyapatite coating [29] (BoneMaster, Zimmer Biomet, Warzaw, IN, USA).

### Surgery

All surgeries were performed by or under direct supervision of an orthopedic surgeon. The procedure was performed under general anesthesia with the leg under traction using an approximately 10 cm lateral thigh incision and with the vastus lateralis muscle held anteriorly. A k-wire was placed using a 135° guide and fluoroscopy to aim for a central femoral head position in both the anterior–posterior (AP) and lateral (LAT) plane. After reaming the implant canal, 6 1-mm tantalum beads were inserted in the femoral head/neck fragment, and 7 tantalum beads were inserted into the greater and lesser trochanter with an 18 cm long bead gun (Wennbergs Finmek, Gunilse, Sweden). Randomization was done after tantalum bead placement. Subsequently, the screw length was measured and the screw and a 4-hole plate was inserted. The wound was closed using resorbable 2–0 suture in the fascia and subcutaneous tissue and nylon 4–0 skin suture. Post-surgery, patients were mobilized with full weight bearing and walking aids as needed. Cefuroxime (Braun, Frederiksberg, Denmark) 1500 mg intra-venous (IV) administration was used as preoperative antimicrobial prophylaxis. Low molecular weight heparin (Dalteparin, Pfizer, Ballerup, Denmark) 5000 international units (IU) was used for thrombosis prophylaxis preoperative as needed and 12 h post-operative for 7 days. Blood transfusion was given postoperative upon anemic symptoms and low hemoglobin level (< 6.0 mmol/L).

### Radiostereometric analysis

**Fig. 3:**
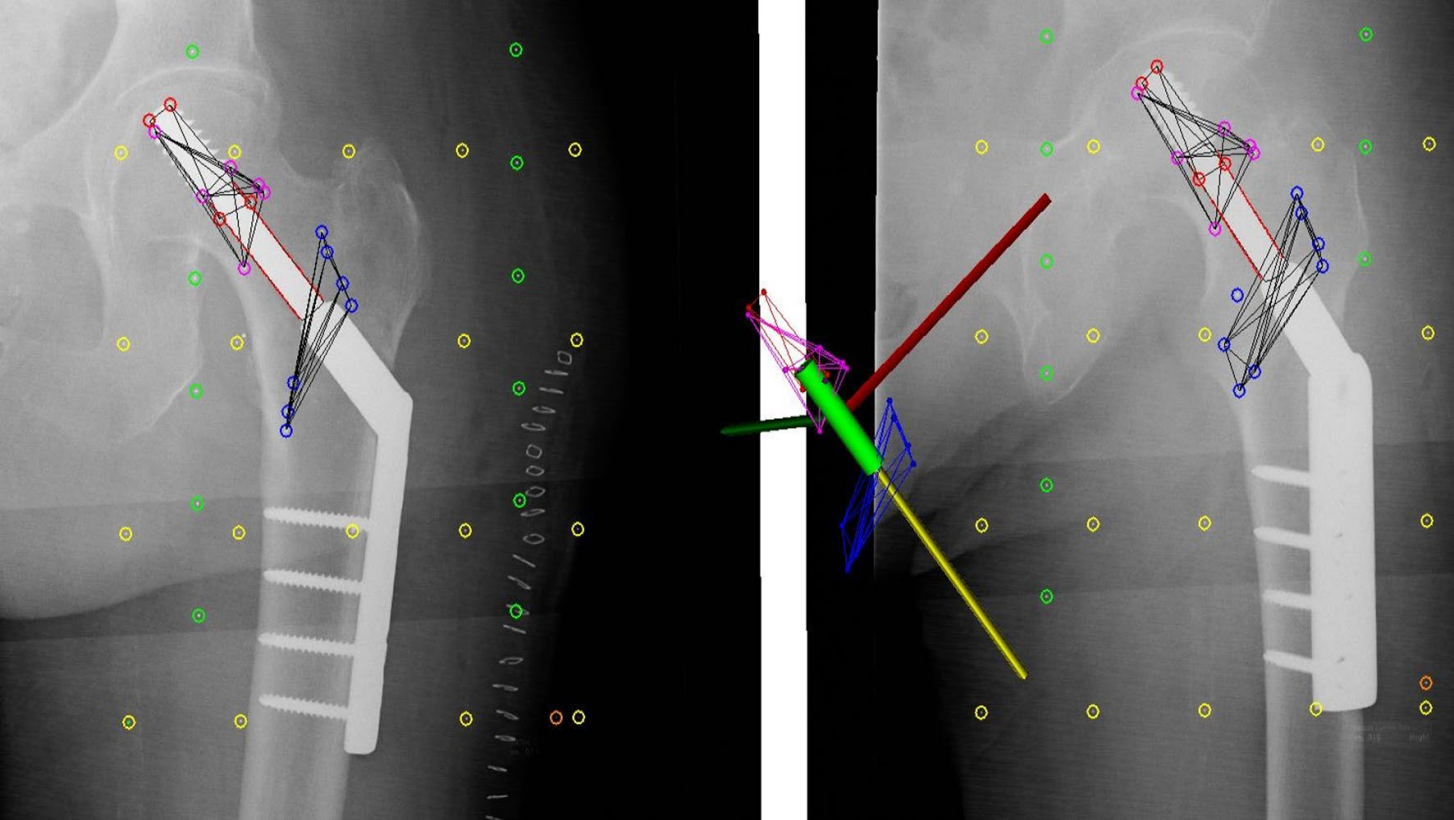
RSA analysis was done marker-based with an EGS cylinder model referenced to the lag screw to align the coordinate system in the screw y-axis (yellow). The x-axis (red) was aligned with the horizontal image plane and the z-axis (green was directed out of plane. The screw markers (red), the head/neck markers (pink), and trochanter markers (blue) were combined in marker-models for each model. The direction of the displayed axes (x, y and z) corresponds to positive values

The first RSA recording was performed within 24 h after surgery and after the patient had been weight-bearing. A standard RSA system with 2 synchronized ceiling fixed roentgen tubes angled 40° towards each other and a uniplanar calibration box (Carbon Box 19, RSAcore, Leiden, The Netherlands) was used [30]. The radiographs were digital and stored in DICOM file-format. RSA recordings were performed with the patients’ legs internally rotated as much as possible to position the sliding hip screw in the horizontal plane, which was also the plane of the calibration box. RSA analyses of the three marker-segments (screw, femoral head/neck, and trochanter) were performed in Model-Based RSA version 4.2 (RSAcore, Leiden, the Netherlands) by an experienced technician blinded to the randomization. Elementary Geometrical Shape (EGS)-RSA was used to apply a cylinder model on the screw to ensure a similar placement of the coordinate system in all patients [31] (Fig. 3). Data for left hips were corrected to right hips to ensure uniform data reporting. Migration of the screw and fracture was expressed as translations along and rotations about the orthogonal x-, y-, and z-axes in the EGS coordinate system of the screw, with the baseline examination as reference. The screw migration with respect to the femoral head/neck segment was described as: x-translation (+ proximal), y-translation (-medial), z-translation (+ anterior), x-rotation (+ posterior screw migration in femoral head), y-rotation (+ posterior screw rotation in femoral head) and z-rotation (+ varus screw migration proximal in femoral head). The fracture stability was described as migration of the femoral head/neck segment with respect to the trochanter region: x-translation (-distal), y-translation (+ fracture impaction), z-translation (+ anterior), x-rotation (+ internal rotation), y-rotation (+ posterior) and z-rotation (+ valgus). The total translation (TT) was calculated as TT = √(*tx*^2^ + *ty*^2^ + *tz*^2^) and total rotation (TR) as TR = √(*rx*^2^ + *ry*^2^ + *rz*^2^).

### RSA precision

The condition number (CN), describing the dispersion of the marker model, was mean 298 (SD 346) in the greater/lesser trochanter, mean 126 (SD 16) for the screw, and mean 120 (SD 61) for the head/neck segment. The rigid body error for marker stability was set at 0.35 mm. When accepting CN higher than 150, the verification of precision is essential [30]. Precision calculations were based on double RSA examinations recorded at 6 weeks, 3 months, or 6 months after surgery.

### Radiographic evaluation

An experienced surgeon classified the pertrochanteric fracture according to Evans classification [32] on preoperative AP and LAT radiographs. Using a DXA scanner a few days after surgery bone mineral density (BMD) measured as T-score was collected. BMD was measured as a total hip score in the non-fractured hip and lumbar spine score (L1-L4), the latter excluding vertebras with fracture collapse. We used the lowest T-score. Post-operatively, at 3-month, and 6-month follow-up AP and LAT radiographs were performed after mobilization with the patient in the supine position and the operated leg internally rotated according to the standardized protocol. The postoperative AP and LAT radiographs were used to calculate the sum of fracture displacement in mm from optimal anatomical fracture reposition (REPO_SUM_) and to measure the Tip Apex Distance (TAD) in millimeters from the tip of the SHS to the apex of the femoral head, which is where the center axis crosses the cortex of the femoral head [11]. The summed distance (TAD_SUM_ = TAD_AP_ + TAD_LAT_) was calculated. Furthermore, the screw position in the femoral head (9 quadrants) was registered according to Cleveland zones [33] on post-operative AP and LAT radiographs.

### Patient reported and clinical outcomes

Patient evaluation was performed pre-operatively asking patients to recall their status before the fracture self-reported mobility level with new mobility score (NMS) (0–9 points) [34], where a score of 9 indicates full mobility and independence and a score of 0 indicates no mobility and maximum dependence, and Harris Hip Score (HHS), where a score of 100 indicates no disability and 0 indicates maximum disability [35]. Furthermore, hip pain at rest (no pain/light—with no activity limitation/mild—not with activity, but high when challenged/moderate—only at daily activity and work, with daily analgesics/severe—strong analgesics and high limitation in daily activities/invalidating pain), walking distance (unlimited/1.5–2 km/0.5—1 km/only indoor/bedridden or wheelchair), use of walking aids (none/cane) – at long distances/always cane)/a crutch/two canes)/walker or two crutches), was collected from the HHS. Cognitive function upon admission was tested with a Danish version of the abbreviated 0–9 mental status test, in which a score of 0–5 is considered low cognitive function [36]. Information regarding gender, age, American Society of Anesthesiology (ASA) score, fracture side, weight, body mass index (BMI), incision length, blood loss, surgery time, surgeon level, screw length, and the hospital of the procedure was collected preoperatively and postoperatively. Six months postoperatively hip range of motion (degrees), leg length difference (cm) with a negative value indicating shortening of the leg with a hip fracture, Trendelenburg sign (positive, horizontal, negative), and Timed Up and Go (TUG) [37] test (seconds) was collected.

### Statistics

Normality of the data distribution was assessed by probability plots. Data following a normal distribution was reported with 95% confidence intervals (CI 95%) and data with non-normal distribution was reported as median values with interquartile range (IQR). Hypotheses of no difference for clinical, patient reported, and radiographic variables comparing the HA-coated group with the NON-coated group was tested statistically using Student’s t-test for normally distributed data Wilcoxon signed-rank test for data with a non-normal distribution. Chi-squared test was used to test categorical data, i.e., screw placement in the femoral head.

RSA measured migration during the follow-up was evaluated as 1) screw migration – with reference to the femoral head/neck segment, and as 2) fracture migration (femoral head/neck segment) – with reference to the trochanter region. Further, fracture migration from baseline to 6 weeks (impaction phase) and from 6 weeks to 6 months (stabilization phase) was compared for all patients. Hypotheses of no difference in screw migration between the HA-coated and the NON-coated group was analyzed using univariate repeated measurement analysis (mixed model) on follow-up time with group HA-coated/NON-coated as fixed effects, and patient as random effect. We used pairwise group comparisons for each follow-up time to describe differences. Unequal standard deviations and correlations of the groups were considered in the analyses. Normal distribution of the mixed-model residuals was tested by Q–Q plots. As no statistically significant or clinically important differences were found between the groups regarding screw migration, the groups were combined for the further statistical analysis (mixed model) of fracture migration during the impaction phase and stabilization phase. Repeatability of RSA double examinations were estimated and reported as absolute mean differences and prediction intervals (1.96 × SD). We used Stata 16 (StataCorp, College Station, TX, USA) for statistical analysis. The statistical significance was set at *p* < 0.05.

### Ethics, registration and funding

The study was approved by the Danish National Scientific Committee on Research Ethics (H-KF-298036, issue date May 3rd, 2006) and Data Protection Agency (2008-41-2757, issue date March 3rd, 2006) and was performed in agreement with the Helsinki ll declaration. The study was registered at ClinicalTrials.gov (NCT05677061)*.* The SHS plate and lag screws including tantalum bead marking were sponsored by Biomet, but the company had no influence on the data analysis, data interpretation or manuscript. The authors declare no conflict of interest.

## Results

### Demographics

The consort study flowchart and the baseline patient demographics are shown in Fig. 1 and Table 1.

**Table 1:** Demographics at baseline

Demographics	HA-coated screw (*n* = 18)	NON-coated screw (*n* = 19)
Gender (*N*), female/male	14/4	17/2
Age (years), mean (range)	80 (57 to 91)	77 (56 to 96)
Fracture side (*N*), right/left	10/8	9/10
Height (cm), mean (CI 95%)	163 (159 to 167)	166 (161 to 171)
Weight (kg), mean (CI 95%)	61 (56 to 66)	61 (55 to 71)
BMI, mean (CI 95%)	22.8 (21.5 to 24.1)	21.8 (18.8 to 24.8)
BMD T-score, mean (CI 95%)	− 3.0 ( − 3.5 to − 2.5)	− 3.1 ( − 3.6 to − 2.6)
Comorbidity (*N*), (ASA I-II/ASA III-IV)	15/3	14/5
New mobility score (*N*), (low 0–5/good 6–9)	5/13	5/14
Cognitive function, median (IQR)	9 (7 to 9)	9 (8 to 9)
Accommodation (*N*), (Own residence/Protected household or Nursing homes)	16/2	16/3
Evans type (*N*), 1/2/4	9/6/3	14/3/2
Surgeon level (*N*), resident/consultant	3/15	6/13
Incision length (cm), mean (CI 95%)	12 (11 to 13)	12 (10 to 13)
Blood loss (ml), mean (CI 95%)	343 (248 to 439)	249 (166 to 331)
Surgery-time (minutes), median (IQR)	66 (55 to 90)	78 (64 to 100)
Screw length (mm), mean (range)	100 (85 to 115)	100 (85 to 115)
Hospital (*N*), Holstebro/Hvidovre	9/9	10/9

ASA score: American Society of Anesthesiology physical classification score system, *N*: number, IQR: interquartile range, CI 95%: 95% confidence interval

### Radiostereometric analysis

Screw migration in the femoral head/neck was similar between the HA-coated group and the NON-coated group at 6 weeks, 3 months, and 6 months (*p* > 0.12) and measures were small (Table 2).

**Table 2:** Screw migration displayed as means in mm and degrees with 95% confidence intervals (CI 95%) at 6 weeks (*N* = 29), 3 months (*N* = 25) and 6 months (*N* = 25) follow-up in the HA-coated screw group and NON-coated screw group

Value/Follow-up		HA-coated screw			NON-coated screw		p-value
	*N*	Mean	CI 95%	*N*	Mean	CI 95%	
*x-translation in mm (*+ *proximal)*							
6 weeks	17	0.51	0.28 to 0.99	12	1.63	− 0.78 to 4.04	0.37
3 months	13	0.49	0.03 to 0.95	12	1.51	− 0.76 to 3.77	0.39
6 months	13	0.46	− 0.03 to 0.95	12	1.49	− 0.91 to 3.99	0.41
*y-translation in mm (-medial)*							
6 weeks	17	0.12	− 0.06 to 0.29	12	− 0.43	− 1.09 to 0.23	0.12
3 months	13	0.06	− 0.15 to 0.27	12	− 0.12	− 0.28 to 0.04	0.19
6 months	13	0.10	− 1.13 to 0.32	12	− 0.06	− 0.19 to 0.06	0.22
*z-translation in mm (*+ *anterior)*							
6 weeks	17	− 0.11	− 0.54 to 0.32	12	− 0.30	− 0.70 to 0.09	0.51
3 months	13	− 0.33	− 0.80 to 0.15	12	− 0.51	− 1.05 to 0.28	0.62
6 months	13	− 0.19	− 0.71 to 0.32	12	− 0.48	− 0.98 to 0.01	0.42
*x-rotation in degrees (*+ *posterior screw migration in femoral head)*							
6 weeks	17	0.10	− 0.93 to 1.13	12	− 1.22	− 3.22 to 0.77	0.25
3 months	13	− 0.83	− 2.01 to 0.34	12	− 1.59	− 3.54 to 0.36	0.51
6 months	13	− 0.08	− 1.08 to 0.93	12	− 1.63	− 3.75 to 0.50	0.20
*y-rotation in degrees (*+ *posterior screw rotation in femoral head)*							
6 weeks	17	1.64	− 1.45 to 4.73	12	1.64	− 1.83 to 5.09	0.99
3 months	13	3.13	− 0.12 to 6.37	12	1.57	− 1.95 to 5.09	0.52
6 months	13	1.51	− 2.16 to 5.18	12	2.59	− 1.40 to 6.58	0.70
*z-rotation in degrees (*+ *varus screw migration proximal in femoral head)*							
6 weeks	17	− 0.21	− 1.06 to 0.64	12	− 1.88	− 6.05 to 2.30	0.44
3 months	13	− 0.2	− 0.93 to 0.90	12	− 1.64	− 5.83 to 2.55	0.46
6 months	13	0.13	− 0.89 to 1.15	12	− 1.43	− 5.79 to 2.94	0.50
*Total Translation in mm (TT)*							
6 weeks	17	1.02	0.51 to 1.54	12	2.16	− 0.26 to 4.58	0.37
3 months	13	1.03	0.47 to 1.58	12	2.12	0.28 to 3.96	0.26
6 months	13	1.13	0.60 to 1.65	12	2.10	0.06 to 4.14	0.36
*Total Rotation in degrees (TR)*							
6 weeks	17	4.74	3.07 to 6.42	12	8.88	4.48 to 13.28	0.09
3 months	13	5.02	2.70 to 7.34	12	8.82	5.49 to 12.15	0.07
6 months	13	5.53	3.24 to 7.81	12	9.35	5.15 to 13.56	0.12

Furthermore, there was no statistically significant difference between groups in fracture translations (*p* > 0.16) and rotations (*p* > 0.08) during follow-ups. Therefore, the two groups were combined (one group) to describe fracture migration (Table 3). At every follow-up statistically significant fracture migration was seen in all directions except for z-translation and z-rotation (Table 3).

**Table 3:** Fracture migration of the femoral head/neck segment relative to the trochanteric region displayed as means with 95% confidence intervals (CI 95%) at 6-week (*N* = 24), 3-month (*N* = 21) and 6-month (*N* = 22) follow-up for all patients (HA-coated and NON-coated groups combined)

Value/Follow-up	All patients		
	Mean	CI 95%	*p*-value*
*x-translation in mm (-distal)*			
6 weeks	− 1.70	− 2.97 to − 0.43	0.01
3 months	− 1.58	− 2.85 to − 0.32	0.01
6 months	− 1.63	− 2.80 to − 0.47	0.01
*y-translation in mm (*+ *fracture impaction)*			
6 weeks	6.18	3.53 to 8.84	0.00
3 months	6.83	4.03 to 9.64	0.00
6 months	7.09	4.09 to 10.09	0.00
*z-translation (*+ *anterior)*			
6 weeks	− 0.42	− 0.02 to 0.19	0.18
3 months	− 0.40	− 0.97 to 0.17	0.17
6 months	− 0.44	− 1.07 to 0.19	0.17
*x-rotation in degrees (*+ *internal rotation)*			
6 weeks	1.78	0.22 to 3.34	0.03
3 months	2.37	0.51 to 4.24	0.01
6 months	2.24	0.36 to 4.12	0.02
*y-rotation in degrees (*+ *posterior)*			
6 weeks	− 3.37	− 5.60 to − 1.14	0.003
3 months	− 3.63	− 6.01 to − 1.26	0.003
6 months	− 3.09	− 5.48 to − 0.70	0.01
*z-rotation in degrees (*+ *valgus)*			
6 weeks	0.96	− 1.61 to 3.54	0.46
3 months	1.27	− 1.62 to 4.16	0.39
6 months	0.58	− 2.11 to 3.27	0.66
*Total Translation in mm*			
6 weeks	7.03	4.25 to 9.81	0.00
3 months	7.65	4.70 to 10.59	0.00
6 months	8.02	4.89 to 11.16	0.00
*Total Rotation in degrees*			
6 weeks	7.20	4.30 to 10.10	0.00
3 months	8.20	4.99 to 11.40	0.00
6 months	7.73	4.69 to 10.77	0.00

The postoperative stereoradiograph constitute the baseline reference for the fracture migration calculations

*Statistical comparison of translations and rotations at 6 weeks, 3 months and 6 months with reference to the baseline examination (day 1)

**Fig. 4:**
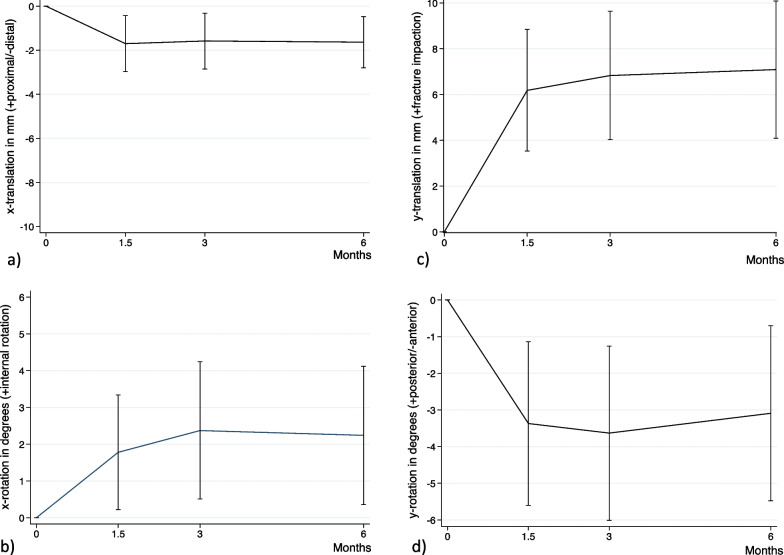
Graphs displaying the mean (CI 95%) fracture migration and rotations of the femoral head/neck segment with reference to the trochanteric region displayed as **a** x-translation, **b** x-rotation, **c** y-translation and **d** y-rotation

Thus, during the impaction phase (the first 6 weeks), the fractures settled with a fracture impaction (y-translation) of mean 5.95 mm (CI 95% 2.87 to 9.04), the femoral head/neck segment rotated anteriorly about the axis of the screw (y-rotation) by mean 2.94° (CI 95% − 5.22 to − 0.66), and the femoral head/neck translated distally (x-translation) by mean − 1.11 mm (CI 95% − 1.87 to − 0.34), with the trochanteric region as reference (Table 4, Fig. 4).

**Table 4:** Fracture migration in mm and fracture rotation in degrees of from baseline to 6 weeks (fracture impaction) and from 6 weeks to 6 months (fracture stabilization)

	Fracture impaction phase		Fracture stabilization phase		p-value*
	Mean	CI 95%	Mean	CI 95%	
x-translation (mm) (-distal)	− 1.11	− 1.87 to − 0.34	− 0.10	− 0.87 to 0.66	0.10
y-translation (mm) (+ fracture impaction)	5.95	2.87 to 9.04	0.89	0.03 to 1.75	0.002
z-translation (mm) (+ anterior)	− 0.09	− 0.82 to 0.63	− 0.02	− 0.52 to 0.48	0.88
x-rotation (°) (+ internal rotation)	1.40	− 0.43 to 3.23	0.27	− 1.19 to 1.72	0.37
y-rotation (°) (+ posterior)	− 2.94	− 5.22 to − 0.66	0.22	− 1.36 to 1.80	0.04
z-rotation (°) (+ valgus)	− 0.12	− 1.93 to 1.70	− 0.58	− 2.30 to 1.14	0.71
Total Translation (mm)	6.60	3.60 to 9.61	0.99	0.03 to 1.95	0.001
Total Rotation (°)	6.33	4.08 to 8.60	0.23	− 3.36 to 3.83	0.002

Only patients with both 6 weeks data and 6 months data are included (*N* = 20)

*Statistical comparison between translations and rotations of the fracture impaction phase and the fracture stabilization phase

During the stabilization phase (6 weeks to 6 months), the fracture impaction (y-translation) was mean 0.89 mm (CI 95% 0.03 to 1.75) and femoral head/neck posterior rotation (y-rotation) was mean 0.22° (CI 95% − 1.36 to 1.80), which was less compared to the impaction phase (*p* < 0.04) (Table 4, Fig. 4).

### RSA precision

For screw migration (*N* = 18), precision (PI 95%) was below 0.33 mm translation in-plane (x- and y-axis), 2.00 mm translation out-of-plane (z-axis), and below 5.78° in rotation about the screw axis (y-rotation) (Table 5). For fracture displacement (*N* = 14), precision was below 2.04 mm translation and below 3.14° rotation (Table 5).

**Table 5:** A. Repeatability of screw migration with reference to the head/neck fracture segment given in the coordinate system of the EGS model of the screw as absolute values from RSA double-examinations (*n* = 18). Combined values for screws with and without HA-coating. B. Repeatability of fracture migration measured as head/neck migration with reference to the trochanter region (as absolute values from RSA double-examinations (*n* = 14). Combined values for screws with and without HA-coating

	Translation (mm)			Rotation (°)		
	*x*	*y*	*z*	*x*	*y*	*z*
*A*						
Mean difference	0.14	0.07	0.48	1.70	2.07	0.58
PI 95% (1.96 × SD)	0.33	0.12	1.98	5.78	5.76	1.61
*B*						
Mean difference	0.18	0.40	0.48	0.88	1.35	0.72
PI 95% (1.96 × SD)	0.62	2.04	1.58	1.93	3.14	2.59

PI = Prediction interval of precision

### Radiographic evaluation

The mean TAD_SUM_ was 20.3 mm (CI 95% 16.5–24.0) in the HA-coated group and 21.7 mm (CI 95% 18.8 to 24.5) in the NON-coated group (*p* = 0.53) (Table 6). Fracture displacement on AP and LAT radiographs combined (REPO_SUM_) was mean 8.2 mm (CI 95% 2.6–13.8) and 5.1 mm (CI 95% 0.26–0.75) in the HA-coated group and NON-coated group, respectively (*p* = 0.28) (Table 6).

**Table 6:** Radiographic measures of screw placement and fracture reduction

Radiographic measures	HA-coated screw	NON-coated screw
*N*	18	19
TAD_AP_ (mm), mean (CI 95%)	9.7 (7.6 to 11.8)	10.4 (9.2 to 11.6)
TAD_LAT_ (mm), mean (CI 95%)	10.5 (8.5 to 12.5)	11.2 (9.0 to 13.4)
TAD_SUM_ (mm), mean (CI 95%)	20.3 (16.5 to 24.0)	21.7 (18.8 to 24.5)
REPO_SUM_ (mm), mean (CI 95%)	8.2 (2.6 to 13.8)	5.1 (2.6 to 7.5)

*N*: Number of patients; TAD_AP_: anterior posterior view; TAD_LAT_: Tip apex distance on lateral view; TAD_SUM_: combined distance of TAD_AP_ + TAD_LAT_; REPO_SUM_: Combined displacement in the fracture measured on AP and LAT radiographs

**Fig. 5:**
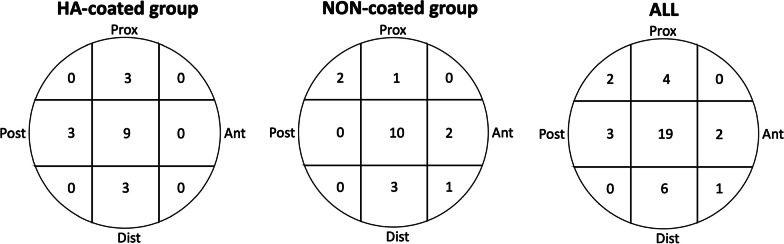
Screw placement in all patients displayed as numbers in 9 quadrants of the femoral head from evaluation on AP and LAT post-operative radiographs. (Ant = anterior, Post = posterior, Prox = proximal and Dist = distal)

The TAD_SUM_ on baseline radiographs did not correlate to screw TT (rho = 0.08, *p* > 0.70) or fracture TT (rho = 0.08, *p* > 0.72) at 6-month follow-up. Fracture reposition (REPO_SUM_) on baseline radiographs correlated to fracture total translation migration (rho 0.65, *p* = 0.002), but not to screw total translation migration (rho = 0.36, *p* = 0.09) at 6-month follow-up. There was no correlation between patient BMD and fracture total translation migration (rho 0.04, *p* = 0.88) or screw total translation (rho 0.06, *p* = 0.79). Screw placement was optimal (central/central) in 19 patients (51%) (Fig. 5.). One patient (NON-coated group, Evans type I, REPO_SUM_ = 0 mm, TAD_SUM_ = 2.17 mm) had a screw in the anterior-distal position of the femoral head, however screw migration was below the mean of both groups (TT = 0.60 mm, TR = 1.32°, y-rotation = − 0.13°), fracture migration was below the group mean (TT = 0.40 mm, TR = 2.29°, x-rotation = 2.26°, y-rotation = -0.26°).

### Patient reported and clinical outcomes

Clinical results are given in Table 7. At 6 months, the walking distance and use of walking aids were similar to the patient reported status before the fracture, while 56% had a positive Trendelenburg test. Nine of 21 patients reported more hip pain after the fracture than preoperative while 12 of 21 reported similar or less hip pain. The worst hip pain reported was moderate.

**Table 7:** Clinical results in patients with baseline (pre-fracture) and at 6-month follow-ups

Clinical data	*N*	Baseline	*N*	6-month FU	Diff	*p*-value^&^
Pain, median, (IQR)	29	1 (1–1)	25	1 (1–2)	*n*.a	0.04
Walking distance (*N*)*	29	8/8/10/3/0	25	7/5/7/6/0	*n*.a	0.07
Walking aids (*N*)^#^	29	13/3/2/0/1/10	25	9/4/3/1/0/8	*n*.a	0.14
Harris Hip Score, (mean, CI 95%)	20	85 (78 to 93)	20	76 (68 to 84)	9 (− 1 to 20)	0.07
Trendelenburg (*N*)^§^			25	14/8/3		
Leg length difference (cm), mean (range)			25	− 0.3 (− 1.5 to 3)		
Timed-Up-and-Go (sec), mean (CI 95%)			21	15 (13 to 18)		
Hip Extension **(**°), median (IQR)			25	0 (0 to 0)		
Hip Flexion, **(**°), median (IQR)			25	110 (90 to 120)		
Hip Abduction, **(**°), median (IQR)			25	45 (30 to 50)		
Hip Adduction, **(**°), median (IQR)			25	30 (30 to 40)		
Hip External rotation, (°), median (IQR)			25	45 (30 to 50)		
Hip Internal rotation, **(**°), median (IQR)			25	25 (20 to 40)		

*Walking distance (unlimited/1.5–2 km/0.5–1 km/only indoor/bedridden or wheelchair), ^#^Walking aids (none/cane)—at long distances/always cane)/a crutch/two canes)/walker or two crutches), ^§^Trendelenburg (positive/horizontal/negative)

^&^Statistical comparison between baseline and 6 months follow-up

## Discussion

Only few studies have assessed screw fixation and fracture migration in pertrochanteric fractures using RSA. The key-finding in this RSA study was similar and low migration of lag screws with HA-coated and NON-coated threads of a SHS in the femoral head/neck fragment in stable (Evans type I, II, IV) pertrochanteric hip fractures. Moreover, fracture migration was observed primarily in the first 6 weeks after surgery as femoral head distal migration, anterior rotation, and fracture impaction.

### Screw migration

In general, HA has been shown to improve osseointegration and fixation with various orthopedic implants including screws [38]. For pertrochanteric hip fractures, Moroni et al. found that a SHS with HA plasma sprayed lag screws with a mean HA-coating thickness of 56 µm was superior to non-coated and reduced the risk of cut-out at 6-month follow-up [18]. These findings were based on evaluation of screw position on postoperative radiographs up to 6 months follow-up including measures of the TAD in a large patient group (only women, *N* = 120). They excluded patients with a proximal screw position in the femoral head (Cleveland proximal zones) and included patients with a TAD > 25 mm (30–35% of the patients) and observed cut-out of the lag screw in 4 patients in the non-coated group (all with a TAD > 25 mm) but in 0 patients in the HA-coated group (regardless of TAD > 25 mm) [18]. The present study that investigated a thinner and electrochemically applied 5 µm HA-coating on the screw thread and utilized a more precise measurement method of screw migration (RSA), could not confirm the superior bone fixation of HA-coated lag screws over non-coated lag screws. At best, there was a tendency of less lag screw migration in the femoral head/neck segment with HA-coating on the screw thread, but this was not clinically relevant and did not lead to failure in terms of screw cut-out. In fact, the screw migrations measured in the present study were at the precision limit of the RSA method. Bojan et al. used RSA to measure the migration of an uncoated lag screw in the cancellous bone of the femoral head in 20 osteoporotic patients with stable pertrochanteric fractures operated with in a short intramedullary nail. They used an anatomical coordinate and point motion of two markers on the screw and reported 6 months screw migration with a mean translation vector of 0.39 mm (range 0.09–3.22) in a patient population similar to our study group [24]. This was less than the mean TT of 1.13 mm in the HA-coated and 2.10 mm and NON-coated screws of the present study. However, the variation in screw migration for NON-coated screws (upper CI 95% TT 4.15 mm) were comparable to the Bojan study. The reason for the good screw fixation and no cut-out failures in both of our study groups might be the stable nature of the fracture with lateral wall support combined with an overall good screw placement in the femoral head. Baumgartner et al. reported that there was a greater risk of screw cut-out with anterior-distal and anterior-proximal screw placement in the femoral head [11]. Gargano et al. supports a central placement in the femoral head to be the most optimal, because the centre of the head has a high bone volume that allows a better anchorage of the screw [39]. Only one of the lag screws in the present study was placed in this sub-optimal position (anterior/distal) but the screw did not cut-out. This supports the importance of optimal screw placement within the femoral head when treating pertrochanteric fractures.

### Fracture stability

In the treatment of pertrochanteric fractures a stable internal fixation accomplished with dynamic compression of the fracture sites is important to achieve stability and healing. Van Embden et al. described a mean shortening of 7.1 mm (range 4.6–10.7) after 6 weeks in 4 pertrochanteric fractures treated with a SHS [10]. Likewise, we found greatest fracture displacement in the first 6 weeks (fracture impaction phase) with mean 5.95 mm (up to 22.57 mm) femoral head/neck impaction in the trochanter, mean 1.11 mm femoral head/neck distal translation, and mean 2.94° femoral head/neck anterior fracture rotation about the screw. Lustenberger et al. described an association between rotation of the femoral head and screw cut-out in patients with pertrochanteric fractures [22]. They found that fracture impaction, the rate of cut-out, and delayed union was significantly higher in patients with rotation of the femoral head compared to patients without rotation [22]. In the current study, very small femoral head/neck migrations of mean 0.89 mm impaction in the trochanter and 0.22° rotation about the screw was measured between 6 weeks and 6 months follow-up (fracture stabilization phase). Overall, this indicates fracture healing and good stability after 6 weeks, which is in agreement with findings by van Embden et al. Progressive fracture migration after 6 weeks could be a risk-factor or signal of non-union, osteonecrosis, or screw cut-out [10, 40].

The biomechanical aspects of rotational stability have been debated for several years in patients with pertrochanteric fractures [22, 41]. Since we had beads placed in 3 segments, we were able to measure rotation of the femoral head/neck fragment in relation to both the trochanter and the screw. Both described an anterior femoral head/neck rotation of about mean 3° (up to 6° CI 95%) up to 6 months, which confirm the rotational stability of the SHS system and underline the importance of a strong screw fixation in the femoral head to withstand rotational forces.

Fracture reposition to anatomical position is attempted during surgery but can be difficult. Ragnarsson et al. studied RSA measured migration of displaced femoral neck fractures and found that intermediate fragments increased fracture instability due to poor bony contact, and that ad latum displacement of more or equal to 1 mm on either AP or LAT radiographic projection increased the risk for non-union [27]. This underlines that an anatomical fracture reduction is central for a favorable outcome of the fixation [27]. In support hereof, we found a correlation between non-anatomical fracture reposition measured on standard post-operative hip radiographs and fracture migration (TT) measured by RSA. Low anterior screw position was only seen in one patient (NON-coated group) and did not affect screw migration or fracture migration compared to the group mean.

### Clinical outcomes

At baseline this fragile patient group reported a pre-fracture mean HHS of 85 points, which was lower than the similar aged background population [42]. However, at 6 months follow-up they regained their pre-operative HHS, which indicates good recovery after treatment of the pertrochanteric fracture. Yet, the number of patients with a good preoperative walking distance performance and no need of walking aids was lower at 6 months follow-up, and the number of patients with walking disability and dependency on walking aids increased. Also, more than half of those evaluated at 6 months had a positive Trendelenburg test. Similarly, Ekström et al. reported that patients with stable pertrochanteric femoral fractures (stable types) experienced a deterioration in their walking ability and activities of daily living [43]. However, some natural decline in physical ability is expected with aging in a fragile patient group with co-morbidities. The preoperative NMS was rated between 6 – 9 (good) for the patients in 72% and the TUG test of mean 13 s (all below 20 s) at 6 months follow-up support patients being independent in daily mobility and reflects accommodation in patients own residence [44]. Thus, the general functional standard was likely better for the patients in the present study than for the general patient with a pertrochanteric fracture [45, 46].

### Strengths and limitations

The strength of this study lies in the double-blinded randomized controlled study design, the high precision of RSA as a validated method to measure migrations of both implants and fractures, and the protocolled similarity of the treatment algorithm in both inclusion sites [8, 9]. Limitations include a skewed sex distribution, with more women than men. However, the case-mix is representative of the background population with 30% females and 12% males at risk of an osteoporotic hip fracture [47]. Due to the high precision of the RSA method, the number of needed patients is low in accordance with the ISO standard for RSA, the guidelines for standardization of RSA, and the power calculation. However, like in other studies, osteoporotic bone was a challenge for stability of the tantalum beads inserted in the bone for RSA measurements [10]. Inevitably, in a fragile hip fracture patient cohort co-morbidity and early death in up to 30% in the first few months after surgery can be quite difficult to compensate for during patient inclusion, follow-up, and analysis. Yet, the number of patients in the present study is higher than in any previously reported RSA study on pertrochanteric fractures. However, the heterogeneity of patients may call for larger study groups and can potentially explain the lack of study group differences as a type-II error.

## Conclusion

There was no clinical benefit of hydroxyapatite coating on SHS screw migration in this patient cohort, which may be explained by a good screw placement. Migration of the pertrochanteric fractures stabilized around 6 weeks follow-up and with acceptable fracture displacement and no mechanical failures.

## Abbreviations

AP: Anteroposterior
ASA: American Society of Anesthesiology
BMD: Bone mineral density
BMI: Body mass index
CI: Confidence interval
CN: Condition number
EGS: Elementary geometrical shape
HA: Hydroxyapatite
HHS: Harris Hip Score
IQR: Inter quartile range
LAT: Lateral
*N*: Number
NMS: New Mobility Score
REPO_SUM_: The summed fracture displacement in mm on AP and LAT radiographs
RSA: Radiostereometric analysis
SD: Standard deviation
SHS: Sliding hip screw
TAD: Tip apex distance
TAD_SUM_: The summed tip apex distance (TAD) in mm on AP and LAT radiographs
TR: Total rotation
TT: Total translation
TUG: Timed up and go test

## References

[1] Zielinski SM (2014). The societal costs of femoral neck fracture patients treated with internal fixation. Osteoporos Int.

[2] Maffulli N, Aicale R (2022). Proximal femoral fractures in the elderly: a few things to know, and some to forget. Medicina (Kaunas).

[3] Borgström F (2020). Fragility fractures in Europe: burden, management and opportunities. Arch Osteoporos.

[4] Dansk Tværfagligt Register for Hoftenære Lårbensbrud, Årsrapport 2021. 1. December 2020–30. november 2021.

[5] RIKSHÖFT ÅRSRAPPORT 2022. 2021–2022.

[6] Ban I (2014). Implementing, adapting, and validating an evidence-based algorithm for hip fracture surgery. J Orthop Trauma.

[7] Parker MJ, Das A (2013). Extramedullary fixation implants and external fixators for extracapsular hip fractures in adults. Cochrane Database Syst Rev.

[8] Frandsen CF (2021). Poor adherence to guidelines in treatment of fragile and cognitively impaired patients with hip fracture: a descriptive study of 2804 patients. Acta Orthop.

[9] Palm H (2012). A new algorithm for hip fracture surgery. Reoperation rate reduced from 18 % to 12 % in 2000 consecutive patients followed for 1 year. Acta Orthop.

[10] van Embden D (2015). The stability of fixation of proximal femoral fractures: a radiostereometric analysis. Bone Joint J.

[11] Baumgaertner MR (1995). The value of the tip-apex distance in predicting failure of fixation of peritrochanteric fractures of the hip. J Bone Joint Surg Am.

[12] Davis TR (1990). Intertrochanteric femoral fractures. Mechanical failure after internal fixation. J Bone Joint Surg Br.

[13] Pervez H, Parker MJ, Vowler S (2004). Prediction of fixation failure after sliding hip screw fixation. Injury.

[14] Nordin S, Zulkifli O, Faisham WI (2001). Mechanical failure of dynamic hip screw (dhs) fixation in intertrochanteric fracture of the femur. Med J Malaysia.

[15] Hernigou P (2006). Total hip arthroplasty after failure of per- and subtrochanteric fracture fixation in elderly subjects. Rev Chir Orthop Reparatrice Appar Mot.

[16] Moroni A (2003). Plate fixation with hydroxyapatite-coated screws: a comparative loaded study. Clin Orthop Relat Res.

[17] Moroni A (2001). Improvement of the bone-pin interface strength in osteoporotic bone with use of hydroxyapatite-coated tapered external-fixation pins. A prospective, randomized clinical study of wrist fractures. J Bone Joint Surg Am.

[18] Moroni A (2004). HA-coated screws decrease the incidence of fixation failure in osteoporotic trochanteric fractures. Clin Orthop Relat Res.

[19] Moroni A (2002). Improvement of the bone-screw interface strength with hydroxyapatite-coated and titanium-coated AO/ASIF cortical screws. J Orthop Trauma.

[20] Celik T (2016). Comparison of the lag screw placements for the treatment of stable and unstable intertrochanteric femoral fractures regarding trabecular bone failure. J Med Eng.

[21] Baumgaertner MR, Solberg BD (1997). Awareness of tip-apex distance reduces failure of fixation of trochanteric fractures of the hip. J Bone Joint Surg Br.

[22] Lustenberger A, Bekic J, Ganz R (1995). Rotational instability of trochanteric femoral fractures secured with the dynamic hip screw. A radiologic analysis. Unfallchirurg.

[23] Bojan AJ (2015). Three-dimensional bone-implant movements in trochanteric hip fractures: precision and accuracy of radiostereometric analysis in a phantom model. J Orthop Res.

[24] Bojan AJ (2018). Trochanteric fracture-implant motion during healing—a radiostereometry (RSA) study. Injury.

[25] Finnilä S (2019). Radiostereometric analysis of the initial stability of internally fixed femoral neck fractures under differential loading. J Orthop Res.

[26] Mattsson P, Larsson S (2003). Stability of internally fixed femoral neck fractures augmented with resorbable cement. A prospective randomized study using radiostereometry. Scand J Surg.

[27] Ragnarsson JI, Kärrholm J (1992). Factors influencing postoperative movement in displaced femoral neck fractures: evaluation by conventional radiography and stereoradiography. J Orthop Trauma.

[28] Magyar G, Toksvig-Larsen S, Moroni A (1997). Hydroxyapatite coating of threaded pins enhances fixation. J Bone Joint Surg Br.

[29] Jørgensen PB (2020). Higher early proximal migration of hemispherical cups with electrochemically applied hydroxyapatite (BoneMaster) on a porous surface compared with porous surface alone: a randomized RSA study with 53 patients. Acta Orthop.

[30] Valstar ER (2005). Guidelines for standardization of radiostereometry (RSA) of implants. Acta Orthop.

[31] Jacobsen A (2018). Model-based roentgen stereophotogrammetric analysis using elementary geometrical shape models: 10 years results of an uncemented acetabular cup component. BMC Musculoskelet Disord.

[32] Meinberg EG (2018). Fracture and dislocation classification compendium-2018. J Orthop Trauma.

[33] Cleveland M (1959). A ten-year analysis of intertrochanteric fractures of the femur. J Bone Joint Surg Am.

[34] Parker MJ, Palmer CR (1993). A new mobility score for predicting mortality after hip fracture. J Bone Joint Surg Br.

[35] Harris WH (1969). Traumatic arthritis of the hip after dislocation and acetabular fractures: treatment by mold arthroplasty. An end-result study using a new method of result evaluation. J Bone Joint Surg Am.

[36] Qureshi KN, Hodkinson HM (1974). Evaluation of a ten-question mental test in the institutionalized elderly. Age Ageing.

[37] Kristensen MT, Foss NB, Kehlet H (2005). Timed up and go and new mobility score as predictors of function six months after hip fracture. Ugeskr Laeger.

[38] Søballe K (1992). Tissue ingrowth into titanium and hydroxyapatite-coated implants during stable and unstable mechanical conditions. J Orthop Res.

[39] Gargano G (2021). Zimmer natural nail and ELOS nails in pertrochanteric fractures. J Orthop Surg Res.

[40] Ragnarsson JI, Kärrholm J (1991). Stability of femoral neck fracture. Roentgen stereophotogrammetry of 29 hook-pinned fractures. Acta Orthop Scand.

[41] Schipper IB (2004). Treatment of unstable trochanteric fractures. Randomised comparison of the gamma nail and the proximal femoral nail. J Bone Joint Surg Br.

[42] McLean JM (2017). Normal population reference values for the Oxford and Harris Hip Scores—electronic data collection and its implications for clinical practice. Hip Int.

[43] Ekström W (2009). Quality of life after a stable trochanteric fracture–a prospective cohort study on 148 patients. J Orthop Trauma.

[44] Podsiadlo D, Richardson S (1991). The timed "Up & Go": a test of basic functional mobility for frail elderly persons. J Am Geriatr Soc.

[45] Kristensen MT (2010). Prefracture functional level evaluated by the New Mobility Score predicts in-hospital outcome after hip fracture surgery. Acta Orthop.

[46] Rosendahl-Riise H (2020). Weight changes and mobility in the early phase after hip fracture in community-dwelling older persons. Eur Geriatr Med.

[47] Armas LA, Recker RR (2012). Pathophysiology of osteoporosis: new mechanistic insights. Endocrinol Metab Clin North Am.

